# MRI and PET/CT in the assessment of lymph node metastases in head and neck cancer

**DOI:** 10.1038/s41598-023-46845-y

**Published:** 2023-11-07

**Authors:** Paul Zwittag, Christian Asel, Michael Gabriel, Nina Rubicz, Belinda Bauer, Nikolaus Poier-Fabian

**Affiliations:** 1 Department of Otorhinolaryngology, Head and Neck Surgery, Kepler University Hospital GmbH, Krankenhausstrasse 9, 4021 Linz, Austria; 2 Department of Radiology, Kepler University Hospital GmbH, Krankenhausstrasse 9, 4021 Linz, Austria; 3 Institute of Nuclear Medicine and Endocrinology, Kepler University Hospital GmbH, Krankenhausstrasse 9, 4021 Linz, Austria; 4 Medical Faculty, Johannes Kepler University Linz, Altenberger Strasse 69, 4040 Linz, Austria

**Keywords:** Cancer imaging, Head and neck cancer, Oral cancer

## Abstract

The aim of this study is to present the diagnostic accuracy of MRI and PET/CT in the evaluation of cervical lymph nodes in patients with head and neck cancer (HNC). Data of 114 patients who underwent MRI and PET/CT prior to surgery in the time period between January 2010 and September 2021 in our center is analyzed retrospectively. Histopathological results of surgical preparations serve as the gold standard. The mean time from MRI to surgery is 22.9 (± 18.7) days, and from PET/CT to surgery 21.7 (± 19.9) days. Sensitivities of 80.4% and 80.4%, specificities of 85.7% and 87.3%, PPVs of 82.0% and 83.7% and NPVs of 84.4% and 84.6% are registered for MRI and PET/CT, respectively. 37 false results are further analyzed with respect to side and level of the affected lymph node, as well as intersections of the two imaging modalities. In 29 patients (25.4%), additional findings are described in PET/CT, 7 (6.1%) of which were histologically confirmed to be further malignancies. A combination of both MRI and PET/CT imaging modalities could improve diagnostic accuracy, especially with regard to sensitivity. A notable number of additional findings in whole body acquisition leads to the potential diagnosis of further malignancies.

## Introduction

Imaging of head and neck cancer (HNC) is a difficulty clinicians have to face in daily practice. Particularly, the complex anatomy of this small area of the body, plus a multitude of other possible pathologies make life challenging^1^. Stage at diagnosis predicts survival rates and affects the management of patients with HNC^2^. According to the 8th edition of the AJCC Cancer Staging Manual (TNM 8), the presence of even just one lymph node metastasis (LNM) leads to a significantly higher tumor stage in most entities of HNC^3^. Approximately two thirds of patients initially present with locally advanced disease, including either a large extension at the primary site and/or spread to the cervical lymph nodes^4,5^. Accurate identification and characterization of LNM by non-invasive imaging has critical prognostic significance in patients with recently diagnosed HNC. Data shows a fundamental difference in outcome based on the number of pathologically positive lymph nodes^2,3,5–7^. Furthermore, accurate information about the presence and location of nodal metastases is important in planning appropriate surgery to address all involved sites and avoid unnecessary extension of surgery^7–9^. The surgeon has to decide whether to perform a selective or a modified radical neck dissection^10^. Therefore, the surgeon will take the primary site, the symptoms of the patient and the imaging results into account. Over and above that, precise imaging is necessary in the evaluation of effective treatment response^11,12^.

Cross-sectional imaging modalities rely on size and morphological criteria in the assessment of lymph nodes in HNC. Identification of metastatic lymph nodes with magnetic resonance imaging (MRI) based on nodal size is limited. This was demonstrated by the variable sensitivity and specificity reported in the literature depending on the various size criteria used^5^. The limitations of the sizebased characterization system are obvious to every clinician: metastasis can be present in normal sized lymph nodes, but not every enlarged node is malignant^13^. Currently, advanced functional MRI methods, such as diffusion and perfusion imaging methods, are used to detect LNM^14–17^. Metastasis may be associated with increased cell density leading to alterations in water diffusivity, which can be measured using diffusion-weighted MRI (DWI)^5^. The accuracy may be limited by large voxel size and low signal-to-noise ratio (SNR)^14–16^. Dynamic contrast-enhanced (DCE-) MRI has been used to assess tumor vascularity. A meta-analysis on its application for lymph node assessment found that the overall accuracy of gadolinium-based DCE-MRI for the assessment of nodal metastases is moderate (sensitivity 72% and specificity 87%)^17^. Finally, a multitude of characteristics, alone or in combination, can lead to a suspect finding in MRI.

There is evidence supporting the superiority of positron emission tomography (PET) imaging using ^18^F-fluorodeoxyglucose (FDG) for detecting locoregional nodal and distant metastases compared to CT and MRI in patients with HNC^2,18,19^. FDG-PET was found to have high sensitivity (92–100%) with mixed specificity (77–93%) in detecting nodal metastases^20,21^. A meta-analysis including 24 studies with 1270 patients with newly diagnosed HNC showed a sensitivity of 91% and a specificity of 87% for the detection of regional nodal metastasis of HNC by FDG-PET^22^. But, previous studies comparing the diagnostic accuracy of cross-sectional imaging with PET/CT were conducted with low patient numbers^23,24^ or used different modalities in different patients^25,26^. Studies investigating fusion techniques of PET and MRI found that using these two modalities in combination could improve both sensitivity and specificity for detecting cervical LNM in patients with HNC^5,27,28^. In previous studies, the role of FDG-PET for the detection of lymph node metastasis in patients with HNC was evaluated mainly by comparing imaging findings with surgical or clinical follow-up findings. Histopathological correlation was rarely available^5^. To our knowledge, this is the first study to compare MRI with PET/CT in the assessment of cervical lymph nodes in one and the same cohort of patients in this magnitude.

The purpose of this study is to compare the diagnostic accuracy of MRI and FDG-PET/CT in the assessment of cervical lymph nodes in patients with HNC. Histo-pathological results of surgical specimen are provided to serve as the gold standard in all patients. Sensitivities, specificities, negative and positive predictive values are defined for both imaging methods. Moreover, additional findings in PET/CT are further analyzed.

## Material and methods

### Study design and population

The present study is a retrospective, single center study, including one hundred and twenty patients who had undergone both MRI of the head and neck and FDG-PET/CT. All patients received neck dissection or diagnostic cervical lymph node resection after imaging at Kepler University Hospital Linz between January 2010 and September 2021. The evaluation was approved by the ethics committee of the Federal State of Upper Austria and was conducted according to the Declaration of Helsinki (EK Nr. 1249/2021). The requirement for informed consent was waived due to the retrospective and observational nature of the present study by the Ethics Committee of the Faculty of Medicine of the Johannes Kepler University Linz. All investigators in the study had full access to the datasets used for this analysis. HNC classical meaning, cancer of unknown primary (CUP), malignant melanoma or lymphoma were noted in all patients in the study group. Imaging to assess the presence of cervical lymph node metastases was performed as staging either primarily after diagnosis and before therapy, or secondarily in the follow-up after therapy of other malignancies. Additional findings in PET/CT were defined as results that indicated further investigation. Patients with distant metastasis (Stage IVc) were not included in the study, due to its inherent contraindication for surgical treatment.

### Data collection

Information about patients age at the day of surgery, sex, diagnosis, imaging results, operative data and histological results were retrospectively extracted from patient records. Data acquisition was carried out in accordance with the regulations on data protection. Every case of discordant or unclear results in MRI, PET/CT and/or histology was revised by a radiologist (CA) and an expert in nuclear medicine (MG). Every suspect lymph node in imaging and pathologic lymph node in histology was assigned to a side and a level of the neck according to the Neck Dissection Classification of 2002 by Robbins^10^.

### Image acquisition

Both MRI and PET/CT imaging were performed after diagnosis of head and neck malignancy, or during the post-therapeutic follow-up of other malignancies. MRI was performed with 1.5 Tesla scanners. Common protocols of MRI included T1-turbo spin echo (TSE), short-tau inversion recovery (STIR) and DCE sequences in axial and coronal orientation. Partially DWI was added in the axial orientation.

PET/CT scans were performed using a dedicated Siemens Biograph 40 Truepoint PET/CT scanner (Siemens Medical Solutions, Hoffman Estates, IL, USA). Patients had fasted for at least six hours and blood glucose levels were measured before the injection of ^18^F-FDG to ensure values below 150 mg/dL. ^18^F-FDG was administered 60 min before imaging through a peripheral vein at a dose of 3.7 MBq/kg (0.0001 Ci/kg). Sequential overlapping emission scans of the whole body were acquired in 3D mode at 3 min per bed position using the axial field of the CT scan. Data regarding the diagnostic accuracy of MRI and PET/CT was assessed separately, there was no fusion of the two modalities.

### Surgery

**Figure 1:**
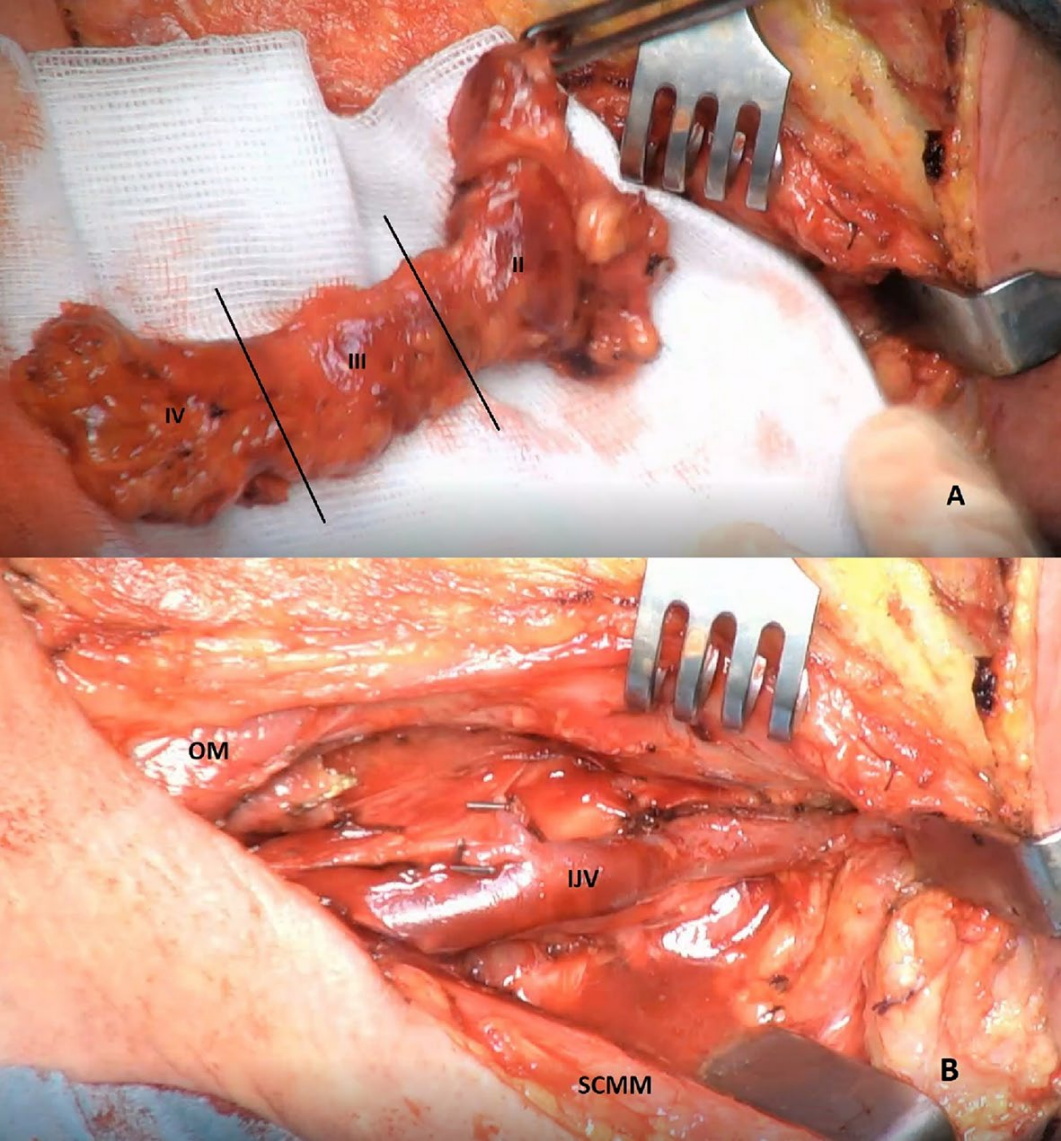
Exemplary intraoperative images of a neck dissection in our center. (**A**) En bloc resected neck dissection preparation with adipose tissue including lymph nodes and lymph vessels of level 2–4 (II, III and IV) on a surgical swab. (**B**) Neck situs from the very same perspective after resection (*OM* omohyoid muscle, *IJV* internal jugular vein, *SCMM* sternocleidomastoid muscle).

In the majority of cases, unilateral or bilateral neck dissection was performed as part of the therapeutic concept of HNC or CUP of the neck. An exemplary intraoperative image of a neck dissection in our center is presented in Fig. 1. In a small number of cases, lymph node resection was performed for diagnostic purposes only. Time from imaging to surgery is displayed in days for each modality. All patients received surgery after image acquisition and the histological result was considered to be the gold standard.

The tumor stage frequencies are presented in Table 1 for all cases of HNC. Patients with elevated risk of lymph node metastasis are defined as having a tumor with a T classification of ≥ 3, lymphatic invasion, grading ≥ 3 and/or p16 positivity.

**Table 1:** Demographic, tumor and imaging characteristics and follow up.

n = 120	Female	Male	Overall
Age in years	63.7 (± 15.1)	60.5 (± 9.3)	61.3 (± 11.1)
Sex	29 (25.4%)	85 (74.6%)	114
Diagnosis			114 (100%)
Squamous cell carcinoma	25 (86.2%)	77 (90.6%)	102 (89.5%)
Cancer of unknown primary	0 (0%)	4 (4.7%)	4 (3.5%)
Others	4 (13.8%)	4 (4.7%)	8 (7.0%)
Tumor stage			108 (100%)
Stage I	12 (41.4%)	16 (20.3%)	28 (25.9%)
Stage II	3 (10.3%)	18 (22.8%)	21 (19.4%)
Stage III	8 (27.6%)	17 (21.5%)	25 (23.1%)
Stage IVa	6 (20.7%)	25 (31.6%)	31 (28.7%)
Stage IVb	0 (0%)	3 (3.8%)	3 (2.8%)
Stage IVc	0 (0%)	0 (0%)	0 (0%)
Elevated risk of LNM in histology of primary site			108 (100%)
No	13 (43.3%)	29 (37.2%)	42 (38.9%)
Yes	17 (56.7%)	49 (62.8%)	66 (61.1%)
Lymph node involvement			114 (100%)
No	15 (51.7%)	48 (56.5%)	63 (55.3%)
Yes	14 (48.3%)	37 (43.5%)	51 (44.7%)
Staging			114 (100%)
Primary	27 (93.1%)	79 (92.9%)	106 (93.0%)
Secondary	2 (6.9%)	6 (7.1%)	8 (7.0%)
Additional finding PET/CT			114 (100%)
No	21 (72.4%)	64 (75.3%)	85 (74.6%)
Yes	8 (27.6%)	21 (24.7%)	29 (25.4%)
Surgery			114 (100%)
ND bilateral	16 (55.1%)	32 (37.7%)	48 (42.1%)
ND unilateral	12 (41.3%)	48 (56.4%)	60 (52.6%)
Lymph node resection	1 (3.4%)	5 (5.9%)	6 (5.3%)
Time MRI to surgery (d)	22.6 (± 19.7)	23.0 (± 18.5)	22.9 (± 18.7)
Time PET/CT to surgery (d)	19.6 (± 16.6)	22.3 (± 21.0)	21.7 (± 19.9)
Metastasis follow up (1 y)			101 (100%)
No	24 (88.9%)	71 (95.5%)	95 (94.1%)
Yes	3 (11.1%)	3 (4.1%)	6 (5.9%)
Duration of follow up (m)	55.0 (± 39.7)	57.1 (± 34.2)	55.6 (± 35.6)

### Follow up

Follow up regime in our center changed over the years. Since 2017 a follow up of 5 years is usual. In the first 2 years of follow up a 3-monthly clinical evaluation is performed. In year 3–5 clinical examination is done in a 6 month interval. MRI of the head and neck is supplemented once a year. In case of advanced cancer or presence of risk factors a CT-scan of the chest and upper abdomen is also performed. Data about the presence of distant metastasis in the 12-month follow up is displayed in Table 1. The duration of the overall follow up and time from surgery to diagnosis of distant metastasis within the first year is given in month. Follow up ended when local relapse or distant metastasis emerged or after 5 years, in patients who were treated after October 2017. Patients, who were treated prior to 2017 partially, are still in livelong follow up.

### Statistical analysis

The obtained data was entered into a dedicated Microsoft Excel spreadsheet. Statistical analyses were performed using SPSS Statistics 25.0 for windows (IBM Corp., Armonk, NY, USA). Descriptive statistics were performed to assess and show frequencies. For nominal variables absolute and relative frequencies are shown. Metric variables are presented with mean value and standard deviation (SD). Cross tables were used to show sensitivity, specificity, positive predictive value (PPV) and negative predictive value (NPV). McNemar´s test was performed to evaluate differences in dichotomous variables. In cases of multiple nominal specifications, the Stuart-Maxwell test was used to evaluate differences between the imaging modalities and the histology. A p-value < 0.05 was considered statistically significant.

## Results

### Characteristics

Patient characteristics, frequencies of malignancies, primary and secondary staging and type of surgery, as well as time from imaging to surgery and tumor characteristics are presented in Table 1.

Results are shown for the whole group and for female and male patients, separately. One hundred and fourteen included patients had a mean age of 61.3 (± 11.1) years at the day of surgery. Twenty-nine (25.4%) of the patients were female, 85 (74.6%) of them were male. One hundred and two (89.5%) patients had squamous cell carcinoma of the head and neck area. Four (3.5%) patients had cancer of unknown primary (CUP)and 8 (7.0%) were subsumed as “other” types of cancer (e.g., adenoid-cystic carcinoma, sinonasal undifferentiated carcinoma, neuroendocrine small cell carcinoma, EBV-associated nasopharyngeal carcinoma etc.). Fifty-one (44.7%) of the patients had pathologically positive lymph nodes in the neck. Twenty-eight (25.9%) patients turned out to have tumor stage I, 21 (19.4%) stage II, 25 (23.1%) stage III, 31 (28.7%) stage IVa, 3 (2.8%) Stage IVb and none had a tumor of stage IVc. 66 (61.1%) patients had elevated risk of LNM in histology of the primary site. In 106 (93.0%) patients imaging was performed as primary staging. In 8 (7.0%) patients the imaging was performed as secondary staging in the follow-up of another malignancy. PET/CT detected further pathologic findings in 29 (25.4) cases. Bilateral neck dissection was performed in 60 (52.6%) patients. Unilateral neck dissection was performed in 48 (42.1%) patients and lymph node resection, only, in 6 (5.3%) patients. The mean time from MRI to surgery was 22.9 (± 18.7) days, the mean time from PET/CT to surgery 21.7 (± 19.9) days.

### Diagnostic accuracy

Cervical lymph node malignancy was confirmed by histology in 51 (44.7%) cases. Imaging with MRI and PET/CT detected suspect lesions in the neck of 50 (43.9%) and 49 (43.0%) patients, respectively.

The sensitivity and specificity of MRI was found to be 80.4% and 85.7%, and the NPV and PPV was 84.4% and 82.0%, respectively. PET/CT had a sensitivity of 80.4%, a specificity of 87.3%, an NPV of 84.6% and a PPV of 83.7% in our sample. McNemar´s test did not reveal any significant differences in MRI vs. histology (p = 1.000) or PET/CT vs. histology (p = 0.815).

### Intersection of false results

**Figure 2:**
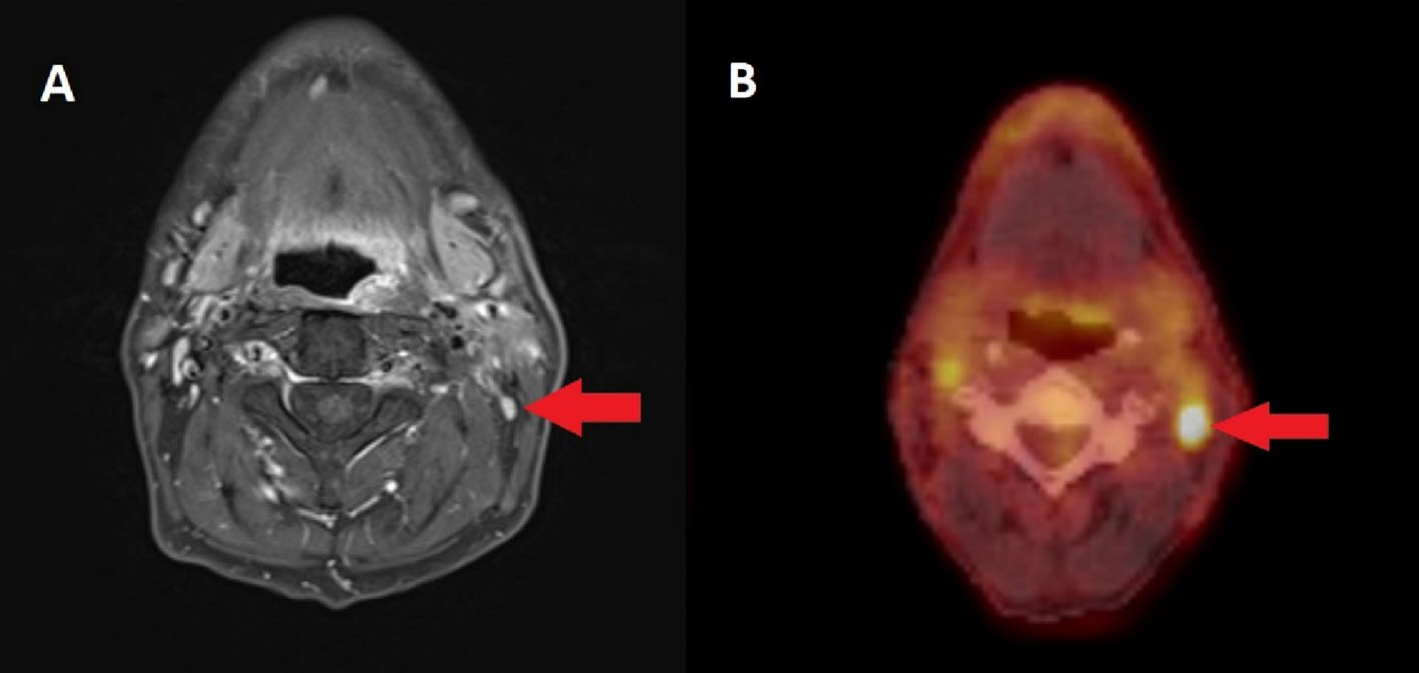
(**A**) MRI of this neck resulted in a false negative finding when (**B**) PET/CT and histological specimen confirmed malignancy. Red arrows mark the region of interest in this case. The time from imaging to surgery was 27 days (MRI) and 33 days (PET/CT). Small volume lesions remain an Achilles heel in the imaging evaluation of lymph node malignancies.

Patients with malignancy in histology were assessed as false negatives in 10 (8.8%) cases by MRI and 10 (8.8%) cases by PET/CT (e.g., Fig. 2). In 7 (6.1%) patients both methods, MRI and PET/CT, yielded a false negative result.

**Figure 3:**
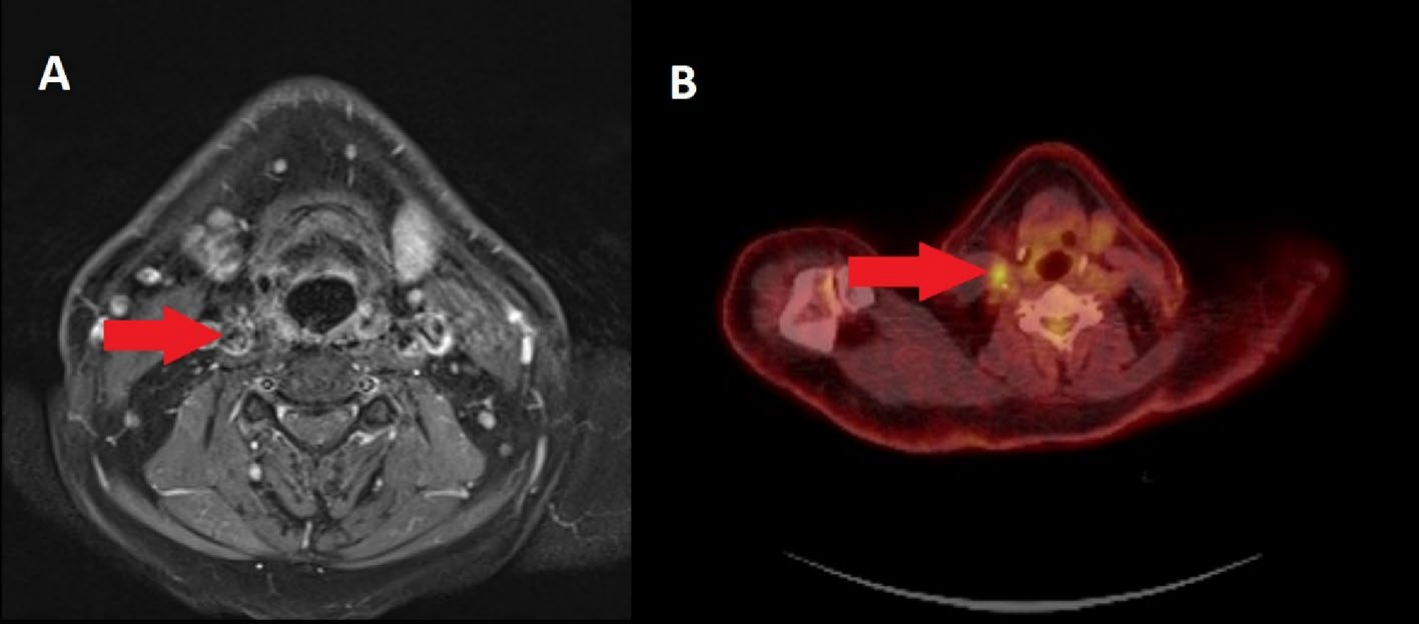
PET/CT showed a false positive result in a negative neck. The area of FDG-uptake (**B**) and the related node in the MRI (**A**) are marked by red arrows. The time from MRI to surgery was 36 days, from PET/CT to surgery 29 days in this case. Inflammation is discussed as a major reason for false positive results in PET/CT.

MRI suspected LNM in 9 (7.9%) negative patients. PET/CT was false positive in 8 (7.1%) patients (e.g., Fig. 3). 4 (3.5%) overlapping false positive findings resulted in 13 (11.4%) false positive results when considering both imaging modalities together.

### Side and location of malignancy

Besides false negative and false positive cases, the side of malignancy was specified correctly in all one-sided cases by PET/CT. In one case, the MRI described suspect lesions bilaterally when malignancy was confirmed for one side only by histology. In 3 cases of bilateral metastases, both methods detected suspect lesions in one side only in the same two patients. The Stuart-Maxwell test showed no significant difference between histology and MRI and PET/CT. P-values were 0.744 and 1.000, respectively.

In the assessment of the level of LNM, one patient with metastasis in level 2 only was classified as having metastases in several levels by MRI. In 3 cases, malignancies were described in level 2 only by MRI, but histology revealed LNM in several levels of one side. PET/CT accordingly suspected malignancies in level 2 in 2 cases with histologically con-firmed malignancies in several levels. In 2 of 3 patients with bilateral LNM, both imaging methods suspected LNM in one side only.

### Additional findings in PET/CT

**Figure 4:**
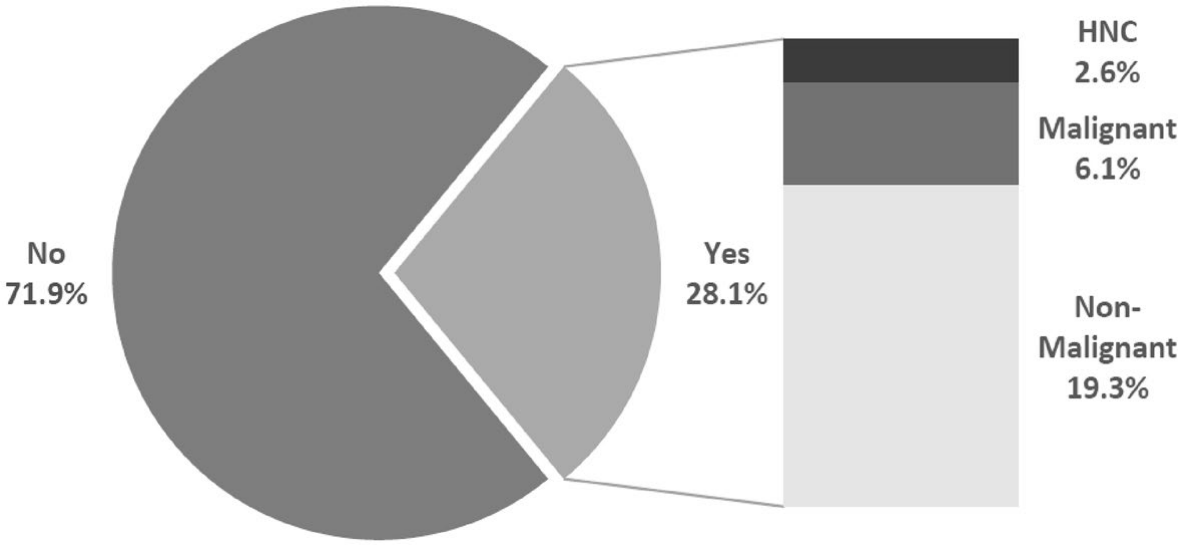
Additional Findings in PET/CT. In 82 (71.9%) patients there was no suspicion described in PET/CT besides HNC. In 29 (25.4%) cases of new diagnosed HNC PET/CT showed an additional finding that led to further investigation. In 7 (6.1%) of these cases the additional finding turned out to be a further malignancy. In 3 (2.6%) further cases PET/CT was performed as secondary staging of another malignant disease and HNC was the additional finding.

In 29 (25.4%) patients, PET/CT showed additional lesions, besides HNC, that led to further investigation. In 7 (6.1%) of these 29 cases further malignant diseases were verified. 5 lung cancers, 1 esophageal cancer and 1 thymoma were proven histologically. In 3 (2.5%) further cases, HNC was initially found incidentally when PET/CT was performed as restaging of other malignancies (Fig. 4).

### Follow up

The mean follow up period was 55.6 (± 35.6) month. Six patients suffered from distant metastases within the first year after surgery. Three of those had local relapse simultaneously. The mean time from surgery to the diagnosis of distant metastasis within the first year was 8 (± 2.4) month. Thirteen (11.4%) patients were lost in follow up within the first year after surgery. Five (4.4%) patients received the follow up elsewhere, 2 (1.8%) patients died within the next few days after the surgery and 6 (5.3%) were lost due to unknown other reasons.

## Discussion

Detection of locoregional lymph node metastases in head and neck cancer is a difficulty that clinicians have to face in every day practice. The presence of LNM may up-stage HNC and worsens the prognosis of the patient. Different imaging modalities are described as complementary problem-solving tools^1,29^. To the best of our knowledge this study is the first to investigate the diagnostic accuracy of MRI and PET/CT in the assessment of cervical LNM of HNC in the very same study population and in a cohort of this size. The results support existing data regarding the diagnostic accuracy of MRI and PET/CT in the assessment of LNM in patients with HNC. Moreover, they reveal both difficulties and advantages involving whole body examinations. In our collective, the sensitivities and specificities of using both modalities, MRI and PET/CT, were found to be between 81 and 88%. Accordingly, Liao et al. describe similar diagnostic accuracy for different modern imaging modalities to define and diagnose cN0 necks. They conclude that avoiding neck dissection and minimizing morbidity is acceptable in some select cases^29^. From a clinical viewpoint, false negative results are problematic in this respect. In our sample, 13 (11.4%) patients had false negative results in at least one imaging modality. In 7 (6.1%) patients, using both MRI and PET/CT gave false negative results. Therefore, the risk of omitting neck dissection still seems to be too high for the majority of patients. However, in select cases where the intraoperative risk of a long surgery is high, a combination of both imaging modalities is favorable, compared to MRI or PET/CT only. Considering our and preexisting data one might even question the use of MRI in the assessment of HNC^30–33^. But from a clinician’s point of view the high resolution of MRI in soft tissue remains indispensable in the planning of the surgical procedure. study by Yamazaki et al. found that true positive nodes in PET/CT had a mean diameter of 13.4 mm, while false negatives had a mean diameter of 3.1 mm. Consequently, the diagnostic accuracy of PET/CT was described as being higher in nodes ≥ 10 mm^31^. Schöder et al. also pointed out that, since nodes < 10 mm make up more than half of LNM, spatial resolution is a limitation of PET/CT with respect to false negative results^32^. An explanation for this can be found in the partial volume effect, a phenomenon that causes degradation of the quantitative accuracy of PET images as a result of the radioactivity spilling over into the background of small lesions < 10 mm in size. This leads to an underestimation of the true standardized uptake value (SUV)^33,34^. Several methods have been developed to correct for the partial volume effect, and have thus significantly improved the diagnostic accuracy of metastatic lymph nodes^35,36^. Nonetheless, false positive results in PET/CT can often be caused by inflammation or lymphoid follicular and parafollicular hyperplasia (Fig. 3)^20,32^.

False negative results concerning the therapy decision and prognosis are more crucial in the assessment of LNMs. Therefore, Chen et al. suggest that MRI measures with a higher spatial resolution can be helpful in detecting small nodes^5^. Functional MRI methods like DWI and DCE-MRI are commonly used in the assessment of lymph nodes. However, it still remains unclear whether a lower apparent diffusion coefficient (ADC)^16,17,37–39^ or a higher ADC^5,14^ indicates metastatic lymph nodes. Chen et al. attribute this to a methodical exclusion of b values below 200 s/mm^2^ of these last two studies, as well as the effect of several other influencing factors^5^. In order to combine data from multiple sites, it is imperative to align data acquisition and analysis methods with respect to cancer imaging^5,40^.

Considering our data, which includes a long study period in a single center analysis, the diagnostic accuracy seems to be good for both MRI and PET/CT. One limitation regarding the comparison of MRI and PET/CT in our analysis is that the findings could be influenced by the results of the examination performed beforehand. As the results of the beforehand performed MRI or PET/CT were not blinded for the examiner of the imaging method performed in second place, the results of the second imaging method could be biased. Ultimately the bias is balanced, due to alternating order of the two methods and very similar time to surgery (Table 1). Nevertheless, it seems that a combination of both imaging methods could reduce false negatives (Fig. 2). Accordingly, preexisting data shows that different modalities supplement each other in the assessment of HNC with regard to both prognosis and decision making in terms of therapy and that PET/CT adds important staging information^1,41^.

**Figure 5:**
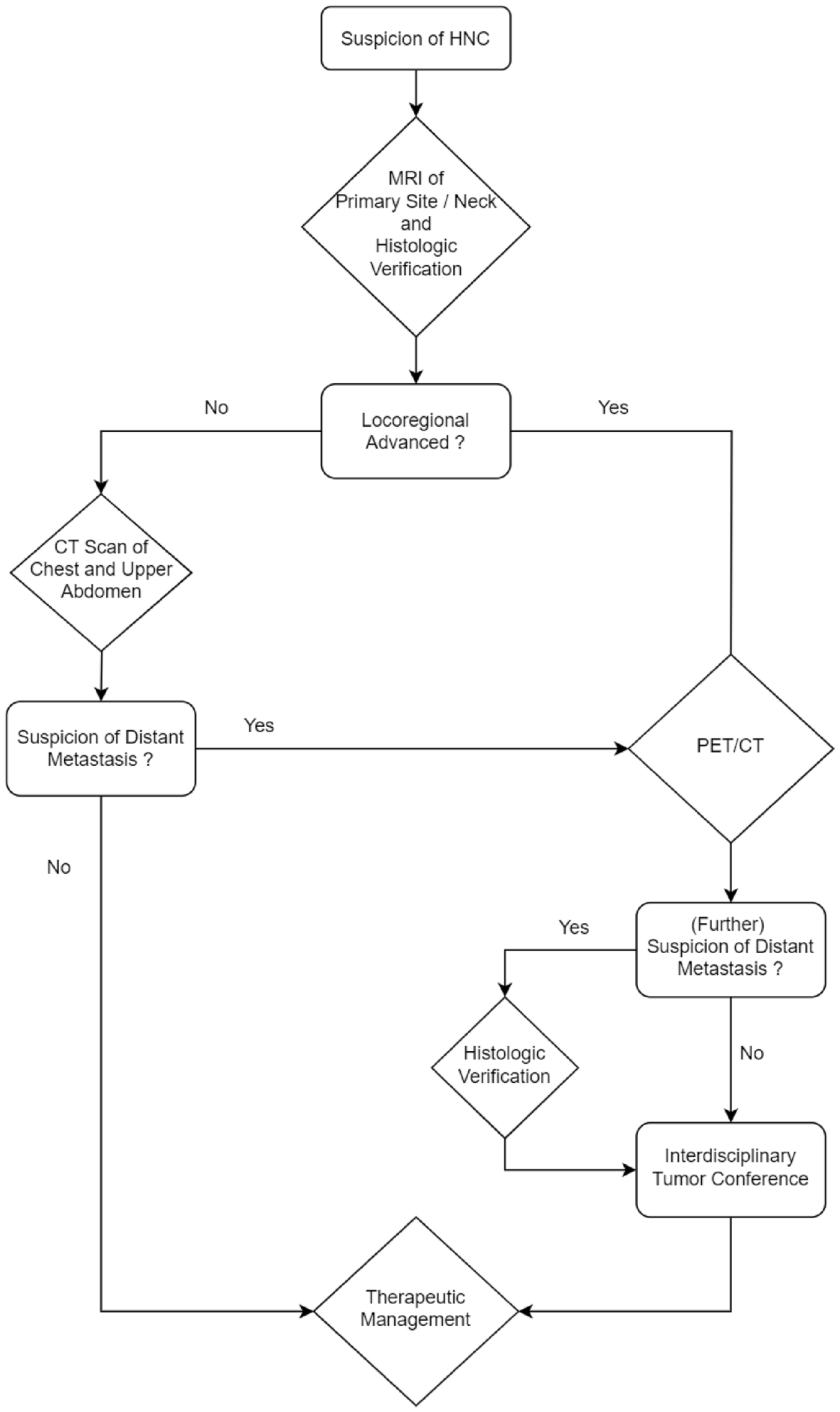
Diagnostic algorithm of HNC in our department. DCE-MRI of the neck is always performed to investigate the primary site and the neck. In early-stage disease staging is completed with a CT scan of the chest and upper abdomen. In cases of locoregional advanced disease PET/CT is performed to assess the presence of distant metastases or further malignancies.

PET/CT is indicated in HNC for the initial workup in large tumors, a clinically positive neck, assessment of treatment response 12 weeks after completion of radiochemotherapy and during follow-up when relapse is suspected^42,43^. A trial by Mehanna et al. showed that surveillance with PET/CT could reduce neck dissection after radiochemotherapy by 80% without impact on overall survival, but with significant cost reduction^44^. Rohde et al. found a significant shift towards palliative treatment decisions in multidisciplinary tumor conferences for recurrent HNC when distant metastases were detected using PET/CT compared to MRI and chest X-rays^45^. Moreover, our data also shows a remarkable proportion of more than a quarter of patients with additional findings via PET/CT. In particular, additional malignancies were found in 6.1% of patients even with PET/CT only. This is an exceptional high number, considering that patients with distant metastases (Stage IVc) were not included in this study, due to contraindication of surgical therapy in these cases. Therefore, we suggest a broad indication for PET/CT not only in recurrent, but also especially in locally advanced HNC, which according to our experience constitutes a large portion of cases (see Fig. 5). Underlining the benefit of parallel use of both methods, there is recent evidence that shows significantly improved prognostic stratification for patients with nasopharyngeal carcinoma through simultaneous use of MRI and PET/CT^46^.

Over and above that, the combined use of PET and MRI by using either software fusion or a hybrid system appears to have synergistic potential for the assessment of metastatic lymph nodes in head and neck cancer^42^. Furthermore, a previous work of our group predicts a major potential of PET/CT, especially for pre-immune checkpoint inhibitor therapy assessment to stratify patients for a maximized therapeutic effect^43^. Both fields are promising for future analyses and need further research.

## Conclusion

In this study we assessed the diagnostic accuracy of MRI and PET/CT in the assessment of cervical lymph nodes in patients with HNC in our center. We were able to register sensitivities of 80.4% and 80.4%, specificities of 85.7% and 87.3%, PPVs of 82.0% and 83.7% and NPVs of 84.4% and 84.6% for MRI and PET/CT, respectively. Our results suggest that a combination of both modalities could improve the diagnostic accuracy, especially in terms of false negative results. Furthermore, whole body acquisition with PET/CT leads to a marked number of additional findings, including further malignancies. Further studies are needed to investigate the synergistic effects of MRI and FDG-PET in the assessment of patients with head and neck cancer.

## Data Availability

Data is available upon reasonable request to the corresponding author.
